# Ambient to Cryogenic High-Frequency Response of Zero-Bias Graphene Photodetectors

**DOI:** 10.1021/acsami.6c05931

**Published:** 2026-06-14

**Authors:** Stefan M. Koepfli, Dominik Bisang, Laurenz Kulmer, Michael Baumann, Shadi Nashashibi, Daniel Rieben, Philippe Peter, Yannik Horst, Yuriy Fedoryshyn, Juerg Leuthold

**Affiliations:** Institute of Electromagnetic Fields (IEF), 27219 ETH Zurich, 8092 Zürich, Switzerland

**Keywords:** graphene, metamaterial, 2D materials, photodetector, cryogenic, nanophotonics

## Abstract

Emerging cryogenic technologies suffer from scaling discrepancies due to limitations stemming from the electrical signal lines connecting the ambient to the cryogenic environment. Currently employed radio frequency cabling offers limited operation bandwidths, therefore requiring large amounts of valuable space and inherently also conducting large amounts of heat. Realizing the ambient-to-cryogenic signal transfer by optical fibers could resolve all of these issues. However, for this purpose, cryogenic-compatible, high-speed, zero-bias-operated photodetectors are required. In this work, we demonstrate a metamaterial-graphene photodetector able to generate high-speed electrical signals in a cryostat without any need for electrical feed lines. This is enabled by a nanostructured metamaterial-graphene architecture that utilizes nanoscale optical hot spots to simultaneously enhance absorption and enable zero-bias carrier extraction. Beyond that, the devices offer high-frequency operations above 100 GHz, a small footprint, and compatibility with various substrates. The signal transfer is further demonstrated by cryogenic optical communication with bit rates of >100 Gbit/s. These results demonstrate a robust platform for high-bandwidth optical-to-electrical conversion, providing a scalable pathway for low-power signal interfacing in next-generation cryogenic systems and quantum technologies.

## Graphical abstract

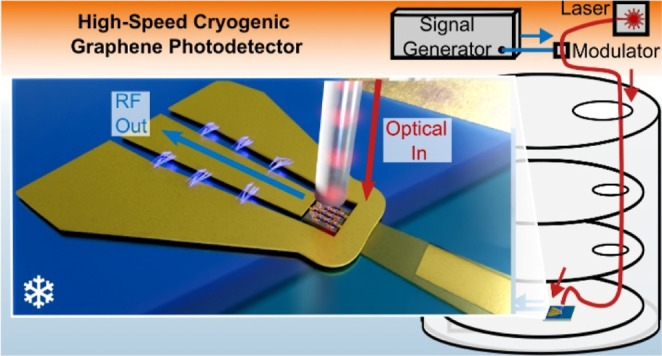

## Introduction

Optical interconnects have established themselves as the dominant solution for data transfer applications at ambient temperatures, as they offer higher data rates and lower losses compared to their electrical counterparts.^1^ In the telecommunications domain, the shift from radio frequency (RF) cabling to optical fibers has been established over the last few decades. With the continued demand for higher bandwidth, data centers and AI centers followed suit and nowadays also heavily rely on optical fibers.^2–6^ More recently, the important emerging quantum applications^7–9^ also target leveraging the same benefits.^10,11^

Currently, RF interconnects are still the main solution for room-temperature (RT) to cryogenic environment signal transfer, which comes with several disadvantages.^12,13^ Typically employed coaxial lines have higher losses at high frequencies, have a much lower operation bandwidth, require attenuation to shield them from thermal noise sources, and also have a larger size and weight compared to optical fibers. One of the most detrimental properties is their inherently high thermal conductivity, which leads to a strong heat transfer from the room-temperature control and readout electronics to the cryogenically operated circuits.^13^ All of these properties either lead to the requirement of larger and more powerful cryogenic refrigerators or ultimately limit the scalability of cryogenically operated circuits altogether.

In recent years, there have been several research efforts targeting the egress data links. Here, coaxial cabling is replaced, and the RF signal is brought out of a cryostat via the optical domain. Several modulator technologies have shown successful optical data transfer with various focuses such as achieving low drive voltages, high bandwidth, and small footprints.^14–21^ Also, the direct modulation of optical emitters has been explored as a solution.^22–24^

As for the ingress data links, where the transfer of an RF control signal to the cryogenic environment is targeted, the state of the art is so far limited. A demonstration showed that it is possible to directly control qubits by employing a commercially available pigtailed InGaAs-based photodiode.^25^ They thereby demonstrated that photonic links are compatible with superconducting qubit operation.

While successful operation was demonstrated, and some photodetectors are able to operate at 4 K,^26–29^ there are still downsides to using conventional photodetectors for cryogenic operation. The bandgap is widening due to cooling, leading to shifts in the maximum operation wavelength, requiring nonestablished material composites for photodiodes.^28^ Further, most high-speed photodiodes require a bias voltage to achieve their high bandwidth. So, either a DC line has to be used for biasing the PD or additional elements have to be added into the cryostat.^30^ And while a DC line is easier to integrate in a cryostat, the induced heat load and size requirements again limit the potential scalability of these systems. Lastly, cryogenic high bandwidth operation beyond 10 GHz of PDs is not explored but could be useful for, e.g., resilient high-frequency qubits.^31^ Therefore, a technology combining zero-bias operation, high bandwidth, and spectral operation in the established communication band is highly desirable to enable the control and scale-up of cryogenic systems. However, such a technology does not yet exist.

In this work, we investigate the high-frequency characteristics of a metamaterial-graphene photodetector from room temperature down to cryogenic regimes. By integrating monolayer graphene into a nanostructured metamaterial absorber, we achieve a zero-bias flat frequency response beyond 110 GHz from room temperature down to 4 K.

We show that cooling the device increases its responsivity, resulting in an RF power gain of >12 dB. To understand the underlying physics, we investigate the interplay between the photovoltaic (PV) and photothermoelectric (PTE) mechanisms on the nanoscale with their subpicosecond dynamics. By this, we can attribute the improvement of the response when cooling to a stronger PV effect, while the counteracting PTE effect is suppressed. To demonstrate the practical impact of the technology, we showcase a cryogenic communication link at 4 K, and we achieve data rates of >110 Gbit/s. Our findings highlight the potential of graphene-metamaterial architectures for scaling future quantum and superconducting systems through high-speed optical interfaces as they operate without any bias circuitry, offer the highest speeds, have no shifts in operation spectrum, and do not suffer from carrier freeze-out.

## Results and Discussion

### Metamaterial-Graphene Photodetector Concept

The envisioned use-case scenario of this work is illustrated in Figure 1a—the ingress data link with a transfer of a radio frequency (RF) signal from room temperature to the cryogenic environment. Classically, semirigid cabling is used as illustrated in Figure 1a, on the left side. Such a refrigerator architecture leads to the following properties:^13^ Typical semirigid cabling has a useful bandwidth of ∼10s GHz, induces a thermal load of ∼1 to 100 mW, has diameters in the mm range, and has a weight of 10 g/m. Furthermore, due to thermal noise, several filters and attenuation stages are employed, thereby adding more components to the refrigerator. At RT, RF signals with mW power levels have to be generated to achieve sub nW signals in the cryogenic environment.

**Figure 1:**
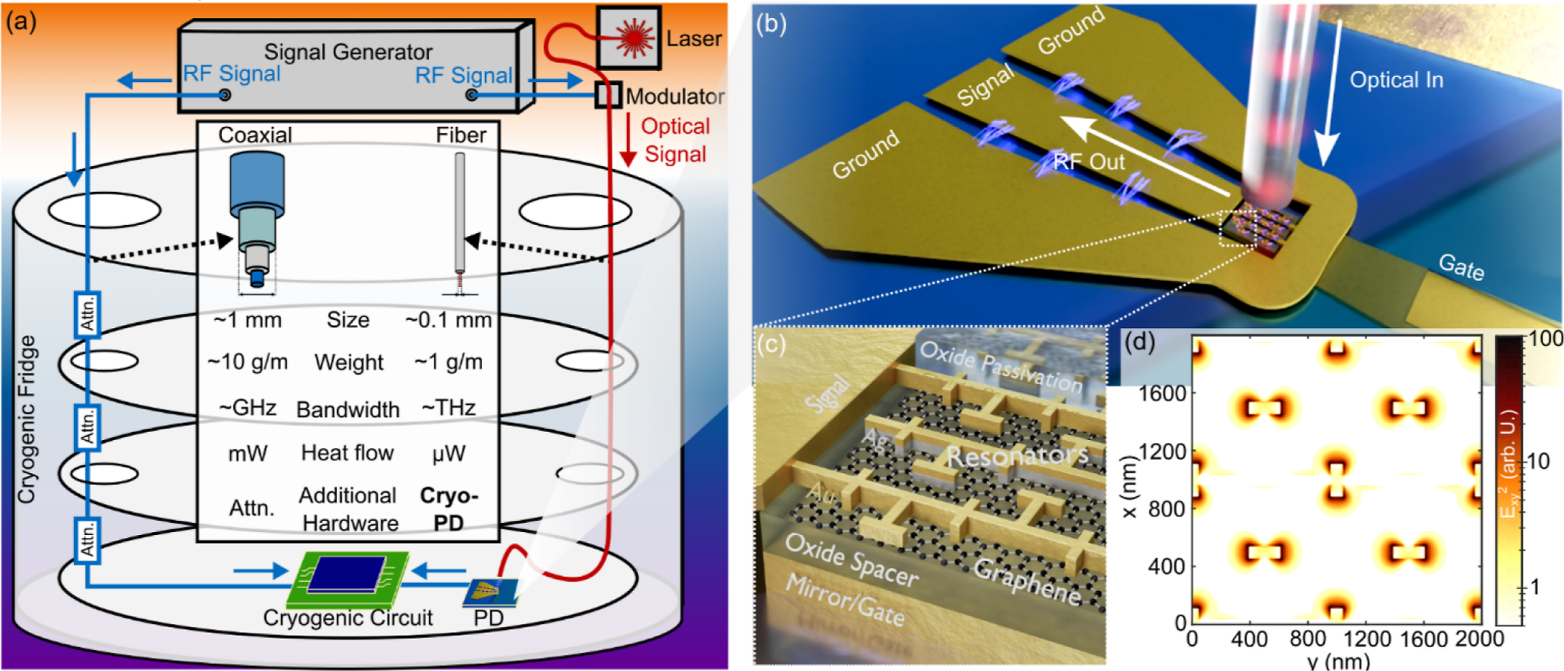
Room temperature to cryogenic environment ingress high-speed link. (a) Envisioned use-case scenario comparison. A radio frequency (RF) signal is to be transferred from a room temperature signal generator down to the cryogenic environment. The left-hand side illustrates the conventional link with a coaxial cable and its properties. The right-hand side illustrates the here-proposed route, where the RF signal is transferred onto an optical carrier guided in an optical fiber with its advantageous properties outlined. In the cryogenic environment, the RF signal is retrieved by a zero-bias-operated photodetector (PD). (b) Artistic visualization of the proposed photodetector illustrating the direct illumination from a single-mode fiber and the RF pads. (c) Zoom-in and cross-section of the graphene-metamaterial PD layer stack. (d) Simulated electric field response of the metamaterial showing the dipole-like induced hot spots.

Optical fibers, on the other hand, offer various advantages in such a scenario compared with the electrical counterpart. Size, weight, and bandwidth, as well as thermal conductivity, are all advantageous as outlined in Figure 1a, on the right side. However, for the conversion of the electrical signal to and from the optical domain, additional optoelectronic devices are required. Typically, an external laser and modulator are required to encode the RF signal on an optical carrier. As these components operate outside of the refrigerator, they do not contribute to the heat load. Also, the components are readily available from established classical telecommunication applications. This leaves the photodetector (PD) operated within the cryogenic fridge to retrieve the RF signal to be directly useable in the cooled environment.

We investigate for this purpose a metamaterial-graphene photodetector as schematically depicted in Figure 1b,c. Such an architecture has previously been demonstrated to operate at RT without an external bias in a photovoltaic operation mode and offers high bandwidths in excess of 500 GHz.^32^ The device has an active area of 10 × 10 μm^2^ and is directly illuminated from the top with a lensed single-mode fiber as schematically depicted in Figure 1b. The metamaterial architecture follows the concept of a metamaterial perfect absorber.^33,34^ It consists of a gold backplane, an aluminum oxide spacer layer, a monolayer graphene, and the resonator layer. The spacer layer thickness, resonator thickness, shape, and passivation thickness are optimized with numerical simulations for the given material system to achieve near-unity absorption.^35^

To enable zero-bias operation of the device, the resonator layer in contact with the graphene is crucial. The resonator layer is formed of dipole resonators oriented in vertical and horizontal arrangements to be responsive to both polarizations. Figure 1d illustrates the resonance of the dipole antennas through electrical field simulations with 1550 nm plane illumination. The dipole resonators are further interconnected to function as the collection electrodes. Lastly, they consist of alternating contact metals that are in contact with graphene; every other line consists of silver with gold on top, which connects to the ground, whereas the remaining structures consist purely of gold, which connects to the signal. The work function difference between the metals and graphene induces a contact doping.^36^ Due to the alternating metals, a local p- and n-doping is induced, which leads to the zero-bias extraction of the photoexcited carriers.^37,38^

### Room-Temperature Photodetector Frequency Response

We first tested the PD at room temperature. The characterization of the metamaterial-graphene PD at room temperature is summarized in Figure 2. The setup is schematically illustrated in Figure 2a. We contact the above-explained PD architecture to be used as a three-terminal device.^39^ The contact lines combined with the resonators and graphene form the source-drain channel. Additionally, the gold backplane is designed to function as an electrostatic gate. All room-temperature measurements are performed within a cryogenic probe station under vacuum.

**Figure 2:**
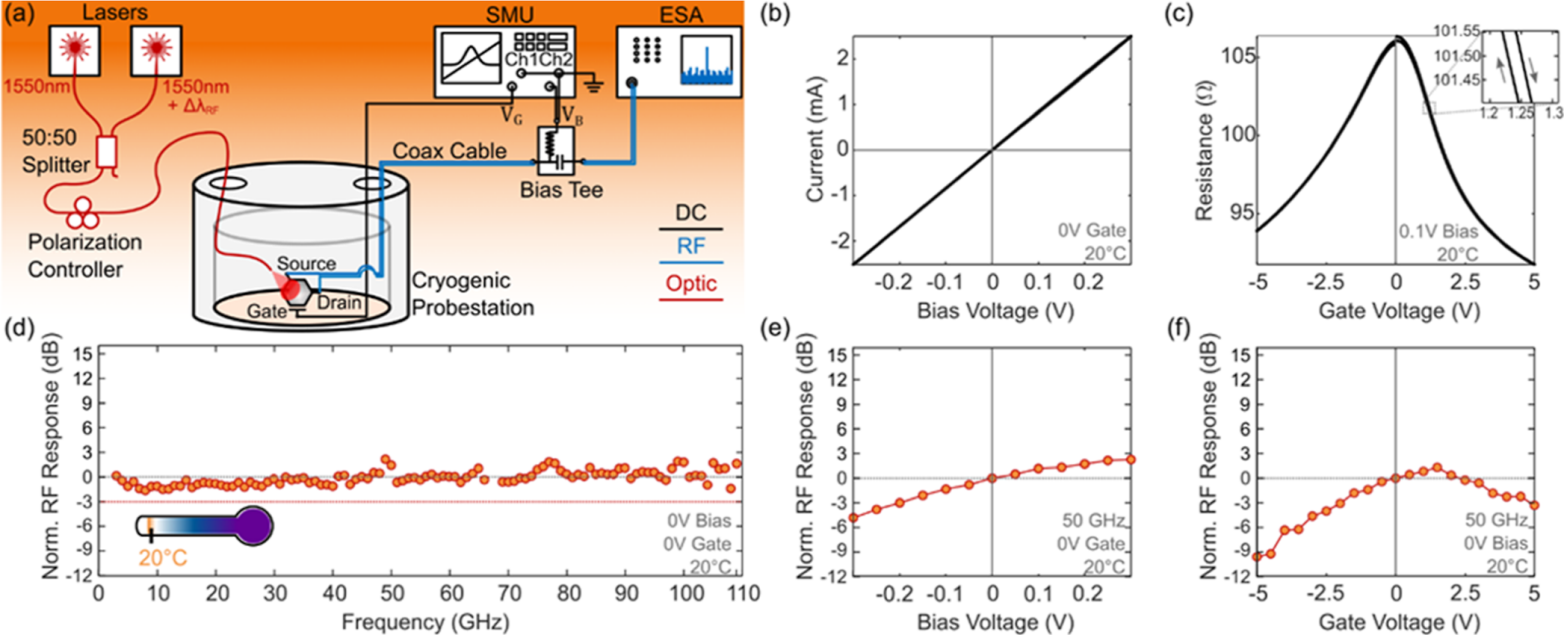
Room-temperature characterization of the photodetector. (a) Schematic setup used for the characterization. The photodetector is placed in a cryogenic probe station. The device is illuminated by using C-band lasers in a laser beating configuration. For the electrical readout, the source-drain channel is connected to a source-measure unit (SMU) and an electrical spectrum analyzer (ESA) where the DC and RF parts are split by using a bias tee. Additionally, the photodetector is operated as a three-terminal device by adding an additional DC line to the gate. (b) IV characteristic of the device showing an Ohmic, low-resistance characteristic. (c) Gate characteristic of the device. The Dirac point is situated close to 0 V, and a small hysteresis is observed. (d) Frequency response of the device in the range from 2 to 110 GHz. (e) RF response at 50 GHz as a function of bias voltage and (f) as a function of gate voltage.

Electrically, the device shows an ohmic characteristic, as shown by the IV measurement provided in Figure 2b. The device resistance at room temperature is 106 Ohms. Applying a gate voltage with a constant bias voltage allows us to characterize a typical graphene field-effect transistor response; see Figure 2c for a back-and-forth sweep. The Dirac point for the device is situated almost at 0 V, indicating a low intrinsic doping level. Furthermore, the two curves overlap well, showcasing the small hysteresis of the device, as seen in the zoom-in.

Next, we tested the frequency response of the device without any electrical bias signals. For this purpose, we use a laser-beating scheme as illustrated in the setup schematic in Figure 2a to generate an RF difference tone. We measure in the frequency range from 2 to 110 GHz and read out the signal with an electrical spectrum analyzer (ESA). Figure 2d shows the frequency response of the metamaterial-graphene PD. We observe a flat frequency response across the entire measurement range with no roll-off characteristics.

Additionally, to understand the PD characteristics with electrical control, we also measured the response at 50 GHz with bias voltage as well as gate voltage by using a source measure unit (SMU). Figure 2e shows the response as a function of bias voltage. A moderate improvement of less than 2.3 dB is possible when positive voltages are applied. Using negative voltages counters the built-in potential of the contacts and reduces the power output. When gating the device, see Figure 2f, the response peaks at 1.5 V with a small improvement of 1.3 dB. The device thereby shows only a minor gain at RT when adding electrical control signals.

### Temperature-Dependent Frequency Response

With the initial characterizations at RT completed, we now cool the devices within the probe station to assess the frequency response at different temperatures, see Figure 3. We choose three distinct temperature points in addition to RT to mimic different operation scenarios: (a) 233 K (−40 °C) mimicking the range where thermoelectric cooling (TEC) is typically achievable. Point (b) is set at 77 K, the boiling point of liquid nitrogen, and last (c) at 4 K, the boiling point of liquid helium. We repeat the same measurements performed at room temperature for these three temperature points. The plots in Figure 3 include the RT measurements from Figure 2d as a reference.

**Figure 3:**
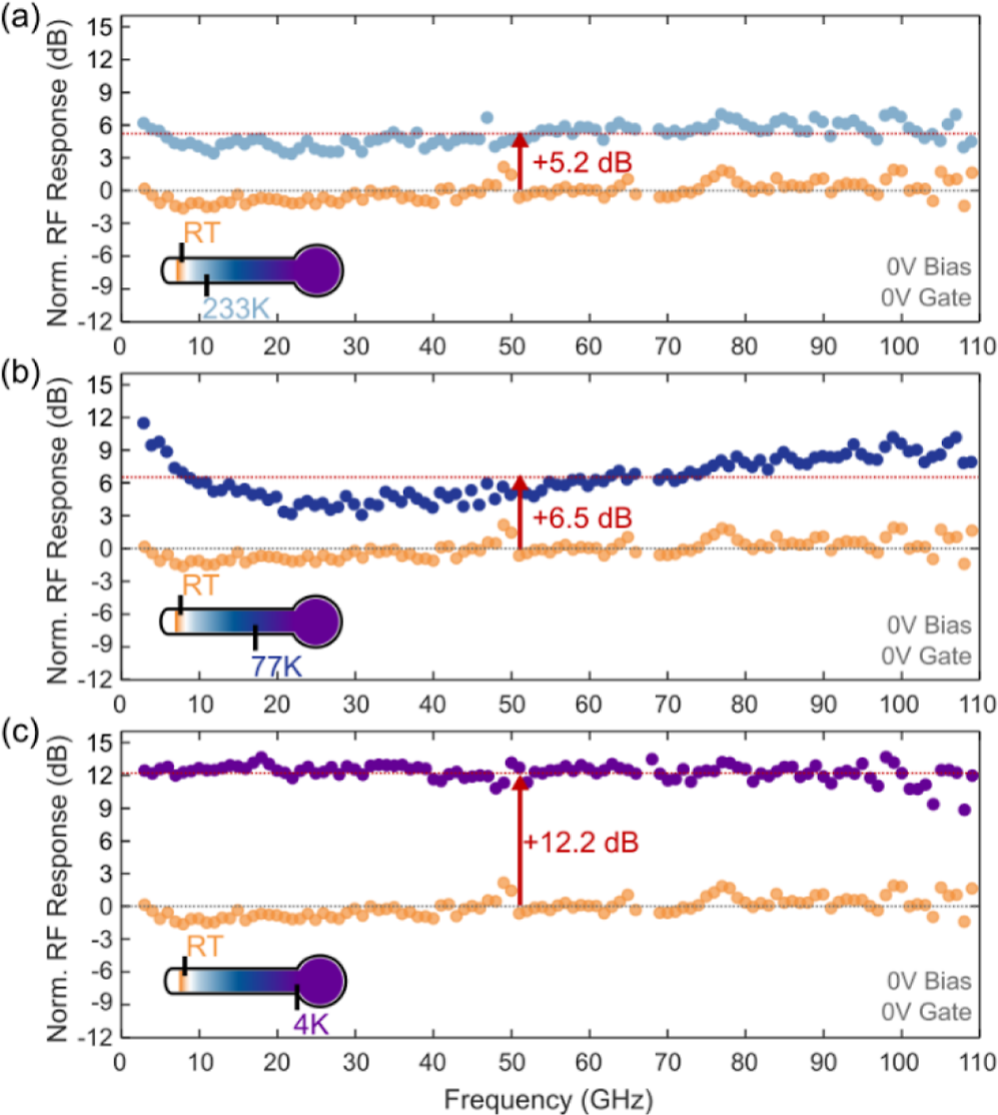
Temperature- and frequency-dependent characteristics. The measured frequency response of the graphene photodetector is between 2 and 110 GHz without any external electrical control. The measurement temperatures are (a) −40 °C, (b) 77 K, and (c) 4 K. Gray data points show RT measurements. Red dotted lines represent the average measured norm. RF response.

The results for (a) 233 K are listed in Figure 3a. We find that the frequency response remains flat and measures an average gain of 5.2 dB (red dotted line) over the measured frequency range in comparison to the RT operation. When the device is cooled further to point (b) at 77 K, see Figure 3b, we observe a continuation of output power gain. Across the frequency response shown in Figure 3b, we measure on average a gain of 6.5 dB. Curiously, the frequency response now has a nonflat behavior. The response drops in the 10s of GHz range and recovers again for higher frequencies, reaching RF outputs close to a 10-fold increase compared to RT. We attribute this frequency-dependent effect to originate from competing photodetection mechanisms that have different temperature and frequency dependences. We discuss this in the following section.

Lastly, Figure 3c shows the measurement results for the device cooled down to point (c) at 4 K, the limit of our measurement setup. The frequency response again returns to a flat response from 2 to 110 GHz. In addition, there is also an increase in the average measured RF power by 12.2 dB. As such, the device shows a high-speed response without any electrical bias and a more than 16-fold increase in output power compared to RT while using the same optical input power. To complete the characterization, we report in the Methods section additionally the gate-dependent RF response and DC responsivity.

To understand the trend of an increasing response with lower temperature and the nonflat behavior, the temperature-dependent carrier dynamics need to be understood. Toward this end, we first present the changes in the graphene characteristics and then continue with a discussion on the temperature dependence of the zero-bias photodetection mechanisms within the device.

### Conductivity and Mobility when Cooling Graphene

In this first part, we explore the conductivity and mobility of graphene when cooling. It is found that the mobility close to the Dirac point increases by a factor of 2.4 when compared against the value at RT. We found these values by evaluating the temperature-dependent electrical DC measurements as follows.

Figure 4a shows the measured conductivity of the device as a function of gate voltage and temperature, illustrated by the solid line. The conductivity increases while reducing the temperature. Additionally, the slope of the conductivity changes with temperature when a gate voltage is applied. To further quantify the effect, we calculate the carrier mobility for the data points when the device is operated with a negative gate voltage. We follow a similar approach as outlined in ref 40.

- First, we transform the gate voltage axis to the induced carrier concentration Δ*n* by applying simplified electrostatic simulations modeling graphene’s response to the gate voltage.^41^ This allows us to relate the conductivity to the induced carrier concentration, which we represent in Figure 4b on a log–log scale. From this figure, we are able to extract the residual carrier concentration *n*_0_.
- In the next steep, we relate the calculated carrier concentration *n* through *n* ^2^ = *n*_0_^2^ + Δ*n* ^2^ to the conductivity, see Figure 4c. The slope of the curves corresponds to the carrier mobility through σ = *qn*μ. The highest slope occurs at low carrier concentrations.
- In the final step, we extract the mobility values for each gate voltage. The extracted values for each temperature point are listed in Figure 4d.

**Figure 4:**
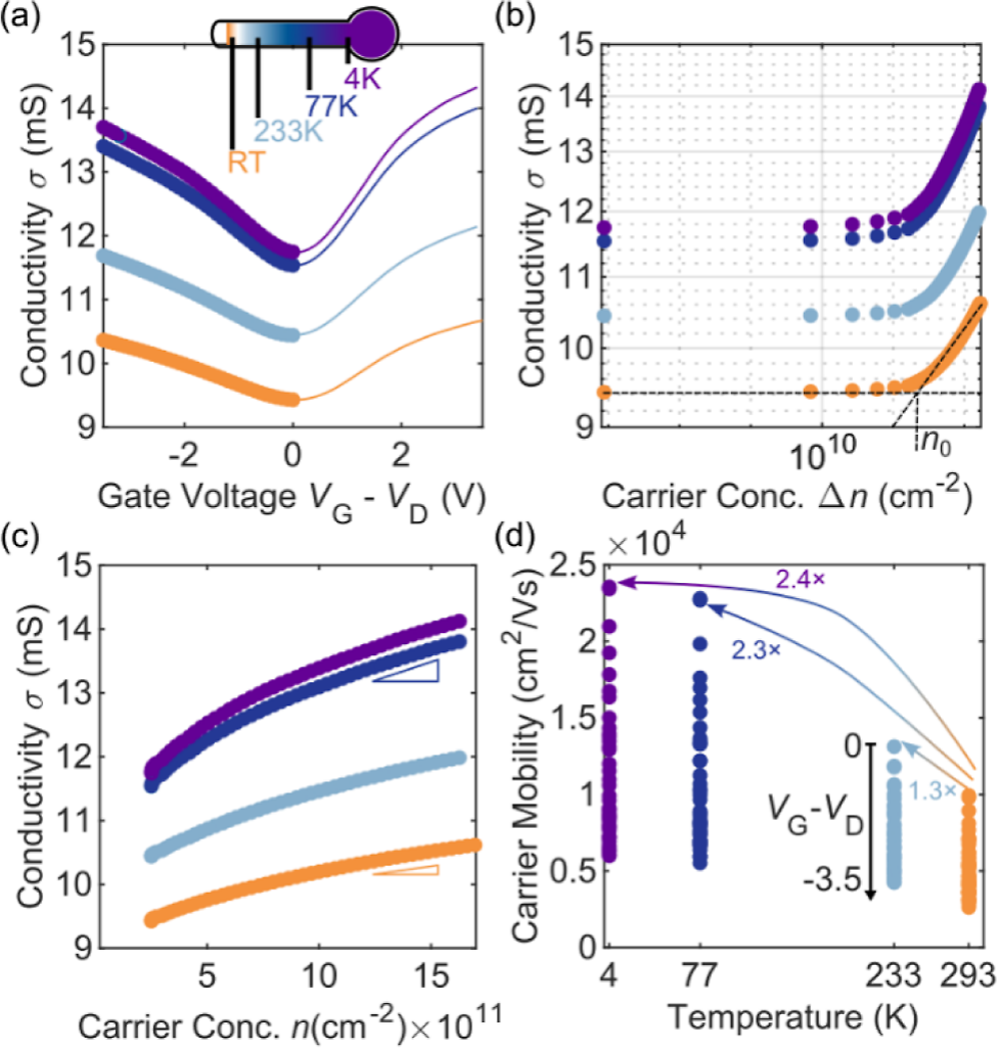
Temperature-dependent gate characteristics and mobility evaluation. (a) Measured conductivity vs gate voltage for the four temperature points. (b) Conductivity vs simulated carrier concentration shift, assuming no residual doping. This allows us to extract the residual carrier concentration. (c) Conductivity vs simulated total carrier concentration. The slope describes the mobility of the device. (d) Calculated carrier mobilities as a function of temperature. Cooling the device from RT to 4 K leads to a 2.4-fold increase in mobility.

The carrier mobility in graphene clearly increases with decreasing temperature, which would benefit the photodetector performance. For the low doping state (i.e., close to the Dirac point), the mobility increases by factors of 1.3×, 2.3×, and 2.4× for 233 K, 77 K, and 4 K, respectively. The increase in mobility flattens out at some point. This is in line with previous reports that showed that the temperature-dependent carrier mobilities of graphene can be modeled with Matthiessen’s rule and are limited through temperature-independent charged impurity scattering.^42–44^ We note that throughout this discussion we neglected the influence of the contact resistance for simplicity.

### Origin of Temperature-Dependent Frequency Response

In addition to the temperature-dependent graphene carrier mobility, we outline the working principle and parameters that are of importance to understand the temperature-dependent frequency response shown in Figure 3.

Beyond the measured change in resistance and mobility, several other parameters within the graphene device change that have an influence on the photodetection process. Zero-bias photodetection within graphene occurs because of asymmetries. Here, this asymmetry lies in the potential landscape induced by metal contact doping. The simulated potential shift at a 0 V gate and bias voltage is shown in Figure 5a. Within this landscape, the two dominant zero-bias mechanisms that occur simultaneously are the photovoltaic (PV) and the photothermoelectric (PTE) effects.

**Figure 5:**
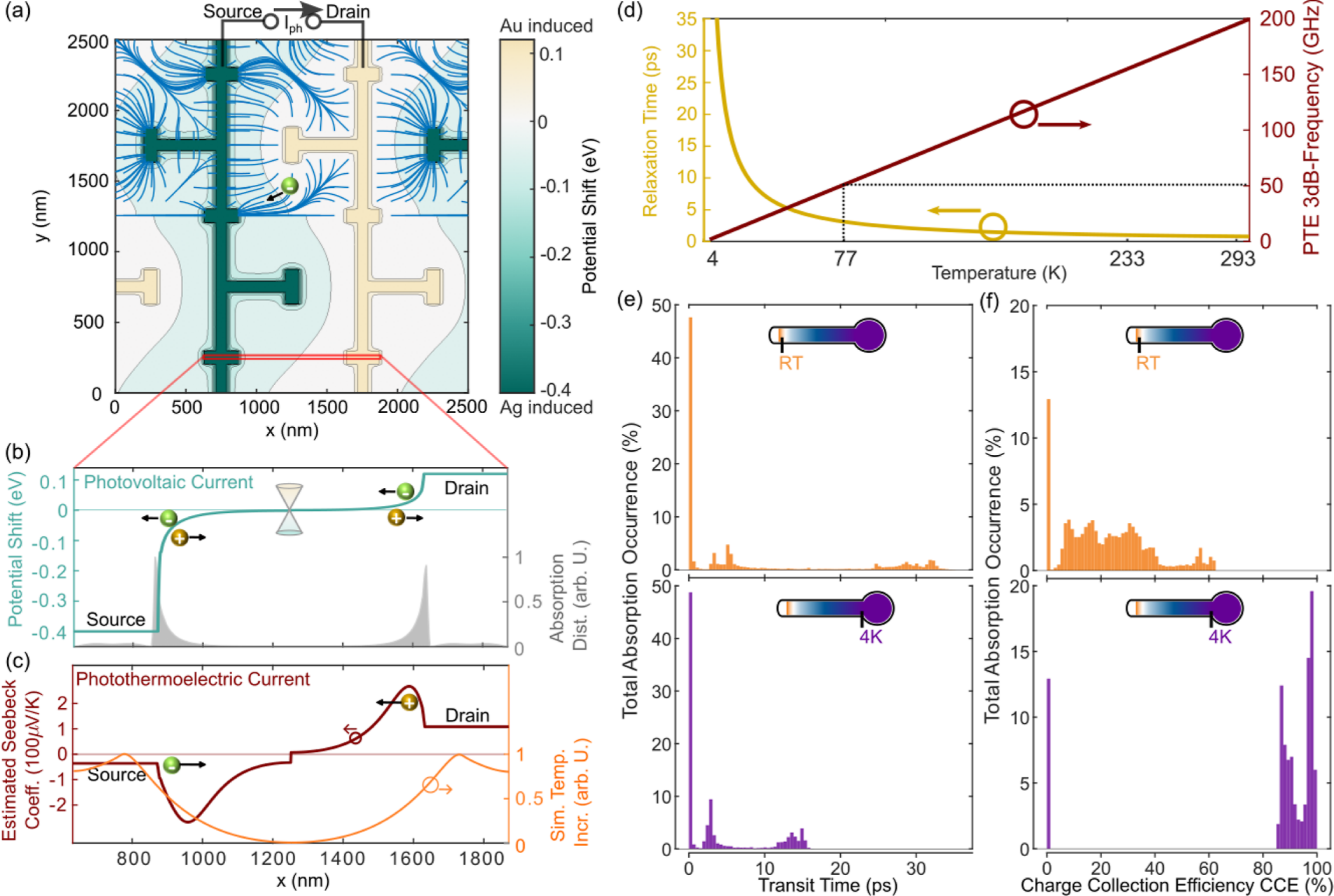
Zero-bias detection mechanisms in the metamaterial-graphene photodetector. (a) Simulated potential shift due to the alternating contact metals at a 0 V gate voltage, where the left contact is silver and the right contact is gold. Blue lines illustrate calculated carrier (electrons) pathways. (b) Cross-section of the potential shift for the source-drain channel along *x* as indicated in (a). Photoexcited carriers are generated by the absorption in graphene, illustrated by the filled gray curve. These carriers (±) are driven to the electrodes due to the built-in potential. (c) Calculated Seebeck coefficient for the same cross-section for the room temperature case (left axis) and the photoinduced temperature change distribution (right axis). Majority carriers diffuse away from local hot spots. (d) Estimated relaxation time based on the super collision cooling model (left axis) and calculated resulting PTE 3 dB frequency (right axis). (e,f) Distribution of simulated carrier transit times (e) and charge collection efficiency (f) grouped by the simulated absorption distribution.

For the PV mechanism, optical excitation creates free charge carriers that are drifting to the collection electrodes due to a built-in potential shift induced by the contact doping, as illustrated in the cross section in Figure 5b. We discuss the efficiency of the PV mechanisms further below after the discussion of the PTE mechanism.

For the PTE mechanism, optical excitation creates localized hot spots, from which majority carriers diffuse away. The efficiency is described by the Seebeck coefficient, which is strongly influenced by charge carrier concentration, mobility, and electron temperature. The calculated Seebeck coefficient (see Methods) for the same cross section in the structure is shown in Figure 5c, on the left axis. The resulting relative increase in temperature for the same cross section is shown on the right axis.

Investigating the interplay of the detection mechanisms, we provide an argument for the temperature dependence of the photoresponse. From the carrier movement depicted in Figure 5b,c, it is evident that the two detection mechanisms oppose each other. We expect this to be the reason for the observed nonflat frequency response in Figure 3b.

To further elaborate, we first look at the frequency characteristics of the two effects. The PV effect is a transit time τ_tr_-dominated mechanism, where the time from generation to collection dominates the frequency response.^45^ The PTE signal, on the other hand, is dominated by the carrier relaxation time τ_rel_.^46^ The relaxation time increases with decreasing temperature, as plotted in Figure 5d, on the left axis. This relation is based on the super collision cooling model.^47,48^

From the relaxation time, we calculate the theoretical 3 dB cutoff frequency for the PTE effect, Figure 5d, on the right axis.

For the first two temperature points—RT and 233 K—the PTE effect retains a frequency response above our setup limit of 110 GHz. The frequency response for operation at 4 K will fall below 1 GHz again outside of our measurement range. Lastly, operating at 77 K will drop the 3 dB bandwidth within our measurement range at an estimated 50 GHz. In addition to the frequency dependence, the PTE has been reported to peak at a crossover temperature of ∼100 K as there is a balance in the Seebeck coefficient with temperature-dependent mobility changes and suppressed electron cooling.^49,50^

Thereby, the combination of change in frequency response and strength of the PTE mechanism could explain the dip in the frequency response that recovers at high frequencies; at 77 K, the PTE has a roll-off characteristic in the measured frequency range and a strong amplitude opposing the otherwise dominant PV effect, leading to the drop in response at low frequency and a recovery of the high response at high frequencies. On the other hand, at 4 K, the PTE is strongly suppressed both in magnitude and in frequency response, thereby returning the device response back to the flat characteristic.

Next, we discuss the PV effect in more detail. We try to explain the gain we observe by modeling the temperature dependence of the PV effect. From the simulated potential shift in Figure 5a, we calculate the carrier pathways within the graphene channel as shown by the blue lines in Figure 5a. By calculating the movement along these paths and the charge that is displaced with respect to the electrodes, we calculated the transit times and charge collection efficiency (CCE). Additionally, we calculate the optical absorption distribution represented by the gray filled curve in Figure 5b on the right axis, which results from the simulated optical resonances shown in Figure 1d.

With the absorption distribution α­(*x*,*y*), transit times τ_tr_(*x*,*y*), and the CCE­(*x*,*y*) for each position (*x*,*y*), one can relate the distribution of photoexcited carriers and their corresponding current generation on a nanoscale. This means we are able to relate how many photoexcited carriers produce how much displacement current, linking absorption to efficiency.

The histograms in Figure 5e show the results of this analysis evaluating the transit time, whereas Figure 5f illustrates the results for the CCE. The panels correspond to the RT and 4 K scenarios. When cooling the device down to 4 K, there are two things happening. First, the transit times get shorter, as shown by the shift in the distribution in Figure 5e. This is due to an increase in carrier mobility (as discussed above) and saturation velocity.^51^ Second, the relaxation time τ_rel_ is getting much longer as discussed in Figure 5d. We model the charge collection efficiency in a Shockley–Ramo-type model^52^ as

1 $$CCE=\int \exp\left(-\frac{\tau_{transit}\left(s\right)}{\tau_{lifetime}}\right)\nabla \psi \left(s\right)ds$$

where ψ is the charge displacement weighting potential and *ds* denotes the line segment along the streamline. The results of this analysis are shown in Figure 5f. Clearly, the charge extraction process is strongly boosted in efficiency for the 4 K case, where the efficiency is moved toward 100%.

From these calculations, a responsivity increase by a factor of ∼3.7× is extracted, which is close to the measured 4.07× corresponding to the measured 12.2 dB gain. The missing factor in the responsivity increase is expected to be a combination of changed electrical properties of the device, the removal of the counteracting PTE mechanism, and a reduction of plasmonic losses due to cooling,^20,53,54^ which would increase the absorbed fraction within the graphene. However, it is difficult to estimate and verify all the absolute values within the device.

### Cryogenic High-Speed Signal Transfer

With the temperature-dependent characterization and modeling of the photodetector finished, we now test it in an RT-to-cryogenic environment signal-transfer scenario.

For this purpose, we tested the metamaterial-graphene PD in a high-speed data experiment. We assembled a setup as schematically shown in Figure 6a. As a signal generator, we use a 256 GSa/s arbitrary waveform generator (AWG) with RF preamplifiers. A plasmonic Mach–Zehnder modulator^55^ (MZM) is used to encode a random bit sequence onto an optical carrier. The random bit sequence signal was used to mimic a relevant high-speed RF signal to be transferred into a cryostat. The optical carrier is set at 1560 nm, fed into the MZM, amplified by an erbium-doped fiber amplifier, and passed through a bandpass filter and polarization controller and then into the cryogenic probe station.

**Figure 6:**
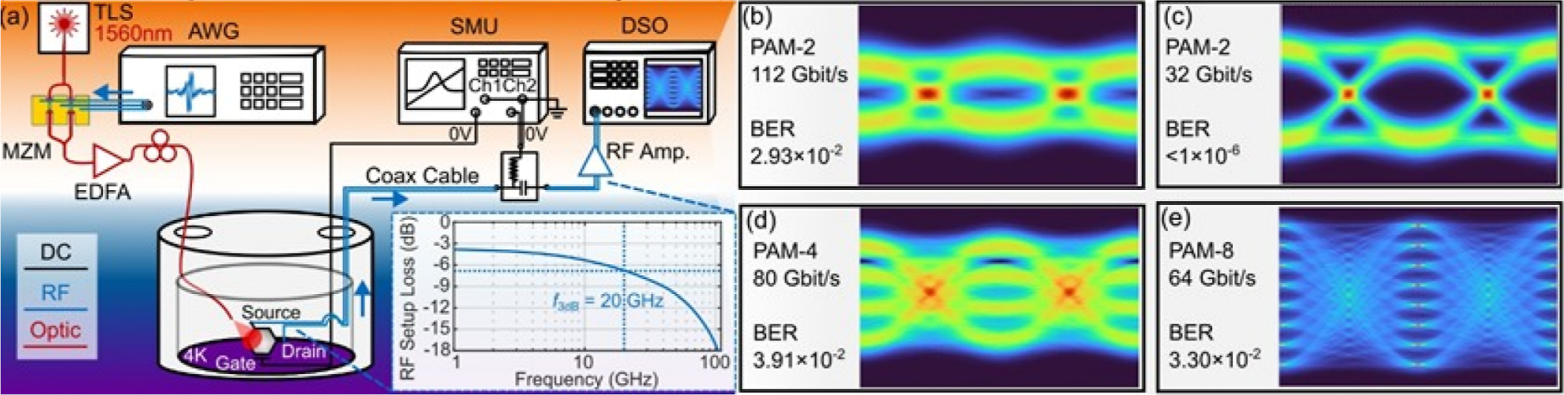
Ambient to cryogenic signal transfer. (a) Schematic simplified setup to transfer an RF data signal via optical fiber to a cryogenic environment. The graphene photodetector is operated at 4 K. The inset shows the extracted RF loss of the RF link from the cryogenic environment to the room temperature measurement equipment. (b–e) Received data signal after offline digital signal processing. The achieved data line rates are (b) 112 Gbit/s and (c) 32 Gbit/s for PAM-2, (d) 80 Gbit/s for PAM-4, and (e) 64 Gbit/s for PAM-8. The provided bit-error ratios (BERs) show successful transmission below the SD-FEC limit.

Within the cryostat, the fiber is positioned over the metamaterial-graphene PD to directly illuminate the device from the top.

In our vision, the PD signal would now be used within the cryostat. Here, we extract this signal from the cryostat to room-temperature measurement equipment for characterization. The signal is then fed to an RF amplifier and an 110 GHz digital sampling oscilloscope (DSO). The electrical interconnect from the cryogenic environment to room temperature suffers from considerable losses at higher frequencies, which we show in the inset in Figure 6a by the curve fitted to the measured loss from calibration measurements. In addition to the high-frequency signals, a source measure unit (SMU) connects to the gate and the source-drain via a bias tee. Both voltages are set to 0 V, and the SMU is only used to keep track of the DC photoresponse for fiber alignment.

The results of the data transfer measurements are shown in Figure 6b–e. Three different modulation formats have been tested: a two-level pulse amplitude modulation (PAM-2) as well as multilevel formats PAM-4 and PAM-8. After DSP, we achieve transmissions below the soft-decision forward error correction (SD-FEC) limit of bit-error rates (BERs) of 4 × 10^–2^. For PAM-2, we achieve data rates of 112 Gbps in Figure 6b; PAM-4 reaches 80 Gbps in Figure 6d and 64 Gbps with the PAM-8 format in Figure 6e. The BERs are also provided in the figures. These data rates are limited by the losses of the RF setup between the cryostat and sampling scope at RT. The 20 GHz 3 dB bandwidth of the RF link strongly decreases the signal generated by the PD. Making use of the signal directly within the cryogenic environment would result in much better signal-to-noise ratios, allowing for higher data rates. However, even with this bandwidth-limited detection scheme, we are able to transfer PAM-2 32 Gbit/s error-free, as shown in Figure 6c. Therefore, the photodetector could already be beneficial for many cryogenic applications.

## Conclusion

In conclusion, we have demonstrated the first high-speed ingress cryogenic data link beyond 100 GHz enabled by a passively operated graphene photodetector. The graphene photodetector does not require any electrical control signals and is shown to have a flat frequency response up to a setup-limited frequency of 110 GHz. When cooling the device down to 4 K, we find a more than 16-fold increase in output power while retaining its high-speed characteristics. We modeled the temperature dependence of both zero-bias photodetection mechanisms, providing an argument for the change in performance. We thereby show that a photovoltaic-operated graphene PD is an ideal candidate to be used as a high-speed electrical signal source within a cryogenic environment. We verify this by performing data experiments showing >110 Gbit/s data transmission, where the main limitation arises from the lossy RF link back to room temperature.

From a theoretical standpoint, further investigating the temperature-dependent frequency response of different graphene devices under different operation conditions could allow for a more detailed understanding of the different mechanisms occurring in graphene photodetectors. From a technical standpoint, this demonstration enables the advantageous implementation of optical fibers without electrical cabling for ingress links in cryogenic refrigerators, which could help to overcome the scaling bottleneck that current refrigerators face.

## Methods

### Device Fabrication

The metamaterial graphene photodetectors were fabricated on standard silicon substrates with a 90 nm silicon oxide layer. In the first step, markers and the gates were deposited using electron beam lithography (EBL), electron beam evaporation, and the lift-off process. The gate layer consisted of 5 nm titanium adhesion layers and 100 nm gold. To create the spacer layer, we deposited aluminum oxide by plasma-enhanced atomic layer deposition (ALD). CVD-grown graphene was procured from Graphene Platform Corp. and transferred with PMMA-supported wet transfer. Using EBL and argon/oxygen reactive ion etching, the graphene channels were patterned. The signal lines including the first half of the metamaterial structures were again deposited using EBL, evaporation, and lift-off. The ground lines and remaining half of the metamaterial were deposited in the same manner. The thicknesses are 30 nm for the gold only half, whereas for the silver contacts, 7 and 23 nm of silver and gold, respectively, were used. The devices were then passivated using 50 nm aluminum oxide deposited in an ALD process. In the last step, the contact pads were opened using photolithography and phosphoric acid wet etching.

To check the influence of the processing on the graphene quality, we performed Raman spectroscopy after the transfer and after completion of the fabrication. Figure 7a shows a typical Raman spectrum after processing. Figure 7b shows the distribution of 2D- and G-peak positions for 625 measurement points recorded over an area of 100 × 100 μm^2^. The processing successfully removed residual doping, achieving low residual carrier concentration with some tensile strain remaining.^56,57^

**Figure 7:**
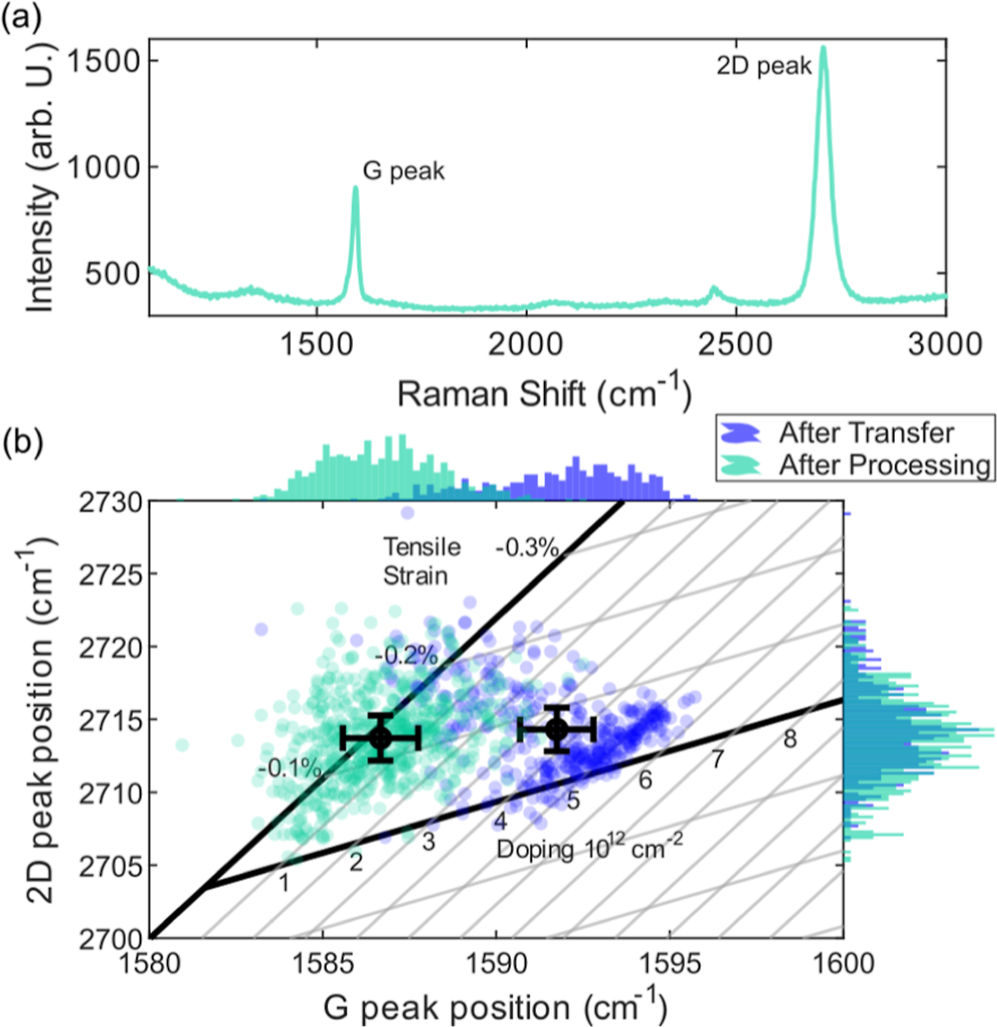
Raman spectroscopy of graphene. (a) Typical observed Raman spectrum showing the G and 2D peaks. (b) Extracted G- and 2D peak positions after the graphene transfer and after processing.

### Graphene Potential Simulations

To simulate the contact doping and applied gate-induced potential shift, we perform 3D electrostatic simulations, solving the Poisson equation on a unit cell of the metamaterial structure. We follow a simplified approach as outlined in ref 41, where we approximate a temperature of 0K to simplify the Fermi–Dirac function.

The setup is as follows: periodic boundary conditions are used for the outer boundaries of the unit cell. The gate electrode is a Dirichlet-type boundary with the value of the applied gate voltage. The graphene is modeled as a Neumann-type boundary. Lastly, the contacts are modeled as Dirichlet-type boundary conditions. The applied values here are calculated by using the analytic formula introduced in ref 36, which leads to 0.12 eV for gold and −0.4 eV for silver.

### Photovoltaic Modeling

In the PV operation mode, one can model the generated photocurrent as charge times carrier generation times collection efficiency. Considering the spatial carrier generation and charge collection efficiency, we describe the generated photocurrent as

2 $$I_{ph}=q\iint \frac{P_{opt}\lambda }{hc}\alpha \left(x,y,\lambda \right)\times CCE\left(x,y\right)dxdy$$

with *P*_opt_ is the optical power, λ the wavelength, and α the position-dependent absorption.

The localized absorption is calculated from electric field simulations performed in CST Studio Suite in the frequency domain. The metamaterial is modeled on a unit cell level with periodic boundary conditions. The structure is excited with a plane with λ = 1550 nm. The absorption is then calculated as $\alpha \left(x,y\right)={\left|E_{xy}\right|}^{2}\sigma_{opt}$ , where |*E*_*xy*_| is the magnitude of the in-plane electric field and σ_opt_ is the optical sheet conductivity of graphene calculated through the Kubo-Greenwood formalism.

As discussed in the main text in eq 1, CCE is a function of the weighting potential ψ and the transit time τ_transit_. ψ is simulated by solving an electrostatic problem where the source and drain electrodes are set to reference potentials of 1 and 0 V, and the conductivity of the graphene sheet is taken as a function of position.^50^ For the transit times, the path along the streamline is segmented in 5 nm steps, and for each step, the localized velocity is calculated. To find the velocity field, first the contact doping-induced potential landscape *U*(*x*,*y*) is simulated (Figure 5a). Taking the gradient of the potential landscape allows us to calculate the velocity field **v**(*x*,*y*) of the photoinduced carriers as

3 $$v\left(x,y\right)=\frac{\mu \left(T\right)\nabla U\left(x,y\right)}{{\left[1+{\left(\mu \left(T\right)\;\nabla U\left(x,y\right)/v_{sat}\left(T\right)\right)}^{2}\right]}^{1/2}}$$

where *v*_sat_(*T*) is the temperature-dependent saturation velocity, which we model as^52^

4 $$v_{sat}=\frac{2}{\pi }\frac{\omega_{OP}}{\sqrt{\pi n}}\sqrt{1-\frac{\omega_{OP}^{2}}{4\pi nv_{F}^{2}}}{\left(\frac{1}{\exp\left(\hbar \omega_{OP}/k_{B}T\right)-1}+1\right)}^{-1}$$

where ℏω_OP_ is the optical phonon energy (∼48 *meV* for Al_2_O_3_)^58^ and *v*_F_ is the Fermi velocity. For the equation to hold, we limit *n*_0_ = 4.25 × 10^10^ cm^–2^. From the velocity field **v**(*x*,*y*) (eq 3), the carrier pathways for both electrons and holes are calculated. The pathways for electrons are illustrated by the blue lines in Figure 5a. Along these paths, for each length segment Δ*s* with direction **s**_**n**_, the transit time is calculated as Δτ_tr_ = Δ*s*/(**v·s**_**n**_) and integrated along the path.

On the basis of this model, the graphene quality plays a crucial role in the photovoltaic performance. Higher mobilities accelerate carriers faster, which reduces the travel time to the collection electrodes. Lower residual carrier concentrations increase the saturation velocities. Both will improve the CCE and the device responsivity.

### Photothermoelectric Modeling

The PTE is described by the Seebeck coefficient *S* and the induced temperature gradient ∇*T*

5 $$I_{ph}\propto -S\nabla T_{e}$$

We therefore need to model the Seebeck coefficient and temperature.

*S* is dependent on the mobility, electron temperature *T*_e_, and the potential landscape *U*(*x*,*y*) as^59^

6 $$S\left(\mu ,U,T_{e}\right)=\frac{2\pi k_{B}^{2}}{3\hbar^{2}v_{F}^{2}}\times \frac{\mu UT_{e}}{\sigma_{\min}+\frac{q}{\pi \hbar^{2}v_{F}^{2}}\mu U^{2}}$$

This Mott formula has been reported to deviate from measured values at certain temperatures,^60^ which makes the quantitative modeling of the PTE difficult in our system.

The induced temperature change Δ*T*_e_ shown in Figure 5c, on the right axis, is calculated by solving the heat equation in a weak heating regime as^60^

7 $$-\kappa_{e}\left(\nabla^{2}\left(\Delta T_{e}\left(x,y\right)\right)-\xi^{-2}\Delta T_{e}\left(x,y\right)\right)=J\left(x,y\right)$$

where κ_e_ is thermal conductivity, ξ = 1 μm is the cooling length, and *J*(*x*,*y*) is the thermal source term, which corresponds to the optical absorption in the graphene. We solve the linearized heat diffusion equation in the frequency domain using a fast Fourier transform (FFT) approach due to the periodic nature of the metamaterial structure.

The PTE response improves with improving graphene material quality. The Seebeck coefficient *S* (eq 6) scales up to a certain point with the graphene mobility,^61^ where there is reduced gain after a certain threshold. Notably, graphene that has low interaction with the local environment is typically able to achieve high mobilities, but cooling times increase, which has a negative effect on the bandwidth of the device as discussed through the relaxation times. The PTE, therefore, typically has a material-based trade-off between response speed and responsivity,^48^ whereas such a limit is not present in the PV effect.

### Gated Photoresponse Measurements

In this section, we add the photoresponse measurement parameters and provide the normalized RF response as a function of gate voltage analogous to Figure 2f for the remaining three temperature points.

The normalized RF power shown in Figure 8a corresponds to an RF tone of 5 GHz and again confirms the observed gain when cooling the device. All curves have been recorded with a 0 V applied bias. The optical power in the fiber inside the cryostat was 10 dBm.

**Figure 8:**
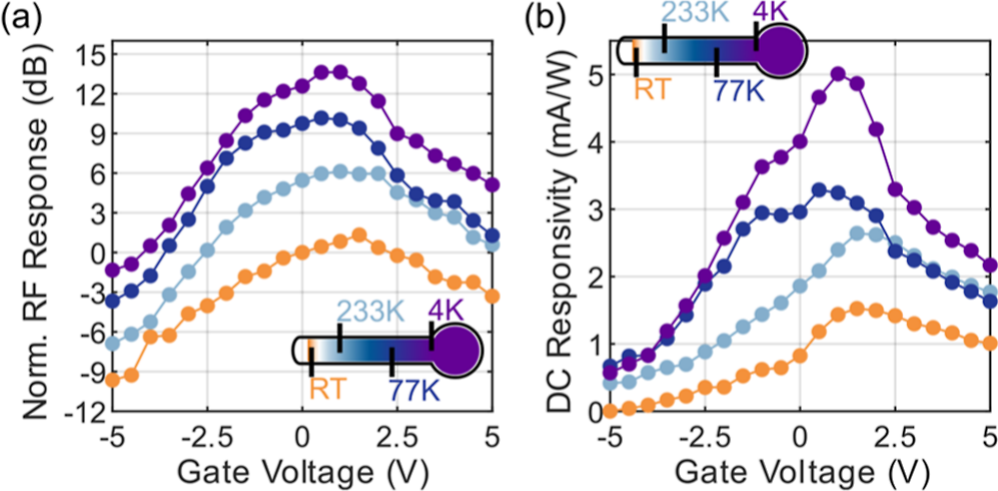
Gated RF and DC photoresponse. (a) Normalized RF power (at 5 GHz) as a function of gate voltage and operation temperature. (b) DC responsivity as a function of gate voltage and operation temperature.

Additionally, we report the DC photoresponses that are extracted from the same measurements as the RF power. The DC photocurrent is measured through the SMU that is connected via the bias tee. Figure 8b shows the DC responsivity as a function of gate voltage and temperature. The curves show the ideal operation point in the range of 0.5 to 1.5 V. The increase in photocurrent matches the measured RF gain; a 12.5 dB gain in RF gain corresponds to a linear factor of 17.78×, which translates to a factor of ∼ 4.22× in current responsivity as *P*_RF_ ∝ *I*_ph_^2^.

## Abbreviations

PD: photodetector
RF: radio frequency
PV: photovoltaic
PTE: photothermoelectric
TLS: tunable laser source
ESA: electrical spectrum analyzer
DSO: digital sampling oscilloscope
AWG: arbitrary waveform generator
RT: room temperature
SMU: source measure unit
MZM: Mach–Zehnder modulator
EDFA: erbium-doped fiber amplifier
DC: direct current
MPA: metamaterial perfect absorber
CCE: charge collection efficiency.

## References

[1] Kikuchi K. (2016). Fundamentals
of Coherent Optical Fiber Communications. J.
Light. Technol..

[2] Cheng Q., Bahadori M., Glick M., Rumley S., Bergman K. (2018). Recent Advances
in Optical Technologies for Data Centers: A Review. Optica.

[3] Baziana P. A. (2024). Optical
Data Center Networking: A Comprehensive Review on Traffic, Switching,
Bandwidth Allocation, and Challenges. IEEE Access.

[4] Butt M. A., Janaszek B., Piramidowicz R. (2025). Lighting the
Way Forward: The Bright
Future of Photonic Integrated Circuits. Sens.
Int..

[5] Daudlin S., Rizzo A., Lee S., Khilwani D., Ou C., Wang S., Novick A., Gopal V., Cullen M., Parsons R., Jang K., Molnar A., Bergman K. (2025). Three-Dimensional
Photonic Integration for Ultra-Low-Energy, High-Bandwidth Interchip
Data Links. Nat. Photonics.

[6] Wan Y., He W., Jaussi J., Liao L., Pan D. Z., Bowers J. E., Rong H. (2026). Integrating
Silicon Photonics with Complementary Metal–Oxide–Semiconductor
Technologies. Nat. Rev. Electr Eng..

[7] Arute F., Arya K., Babbush R., Bacon D., Bardin J. C., Barends R., Biswas R., Boixo S., Brandao F. G. S. L., Buell D. A., Burkett B., Chen Y., Chen Z., Chiaro B., Collins R., Courtney W., Dunsworth A., Farhi E., Foxen B., Fowler A., Gidney C., Giustina M., Graff R., Guerin K., Habegger S., Harrigan M. P., Hartmann M. J., Ho A., Hoffmann M., Huang T., Humble T. S., Isakov S. V., Jeffrey E., Jiang Z., Kafri D., Kechedzhi K., Kelly J., Klimov P. V., Knysh S., Korotkov A., Kostritsa F., Landhuis D., Lindmark M., Lucero E., Lyakh D., Mandrà S., McClean J. R., McEwen M., Megrant A., Mi X., Michielsen K., Mohseni M., Mutus J., Naaman O., Neeley M., Neill C., Niu M. Y., Ostby E., Petukhov A., Platt J. C., Quintana C., Rieffel E. G., Roushan P., Rubin N. C., Sank D., Satzinger K. J., Smelyanskiy V., Sung K. J., Trevithick M. D., Vainsencher A., Villalonga B., White T., Yao Z. J., Yeh P., Zalcman A., Neven H., Martinis J. M. (2019). Quantum Supremacy
Using a Programmable Superconducting Processor. Nature.

[8] Cao Y., Romero J., Olson J. P., Degroote M., Johnson P. D., Kieferová M., Kivlichan I. D., Menke T., Peropadre B., Sawaya N. P. D., Sim S., Veis L., Aspuru-Guzik A. (2019). Quantum Chemistry
in the Age of Quantum Computing. Chem. Rev..

[9] Daley A. J., Bloch I., Kokail C., Flannigan S., Pearson N., Troyer M., Zoller P. (2022). Practical
Quantum Advantage
in Quantum Simulation. Nature.

[10] Han X., Fu W., Zou C.-L., Jiang L., Tang H. X. (2021). Microwave-Optical
Quantum Frequency Conversion. Optica.

[11] Joshi S., Moazeni S. (2024). Scaling up Superconducting Quantum
Computers With Cryogenic
RF-Photonics. J. Lightwave Technol..

[12] Holmes D. S., Ripple A. L., Manheimer M. A. (2013). Energy-Efficient
Superconducting
Computing Power Budgets and Requirements. IEEE
Trans. Appl. Supercond..

[13] Krinner S., Storz S., Kurpiers P., Magnard P., Heinsoo J., Keller R., Lütolf J., Eichler C., Wallraff A. (2019). Engineering
Cryogenic Setups for 100-Qubit Scale Superconducting Circuit Systems. EPJ. Quantum Technol..

[14] Eltes F., Villarreal-Garcia G. E., Caimi D., Siegwart H., Gentile A. A., Hart A., Stark P., Marshall G. D., Thompson M. G., Barreto J., Fompeyrine J., Abel S. (2020). An Integrated Optical
Modulator Operating at Cryogenic Temperatures. Nat. Mater..

[15] Youssefi A., Shomroni I., Joshi Y. J., Bernier N. R., Lukashchuk A., Uhrich P., Qiu L., Kippenberg T. J. (2021). A Cryogenic
Electro-Optic Interconnect for Superconducting Devices. Nat. Electron..

[16] Pintus P., Singh A., Xie W., Ranzani L., Gustafsson M. V., Tran M. A., Xiang C., Peters J., Bowers J. E., Soltani M. (2022). Ultralow Voltage, High-Speed, and
Energy-Efficient
Cryogenic Electro-Optic Modulator. Optica.

[17] Pintus P., Ranzani L., Pinna S., Huang D., Gustafsson M. V., Karinou F., Casula G. A., Shoji Y., Takamura Y., Mizumoto T., Soltani M., Bowers J. E. (2022). An Integrated
Magneto-Optic
Modulator for Cryogenic Applications. Nat. Electron..

[18] Schwarzenberger, A. ; Kholeif, H. ; Kotz, A. ; Kuzmin, A. ; Mertens, A. ; Eschenbaum, C. ; Ramann, G. ; Zyskind, J. ; Lebby, M. ; Randel, S. ; Freude, W. ; Koos, C. First Demonstration of a Cryogenic Silicon Organic Hybrid (SOH) Mach-Zehnder Modulator with a Sub-1V π-Voltage. In Conference on Lasers and Electro-Optics 2023; Optica Publishing Group: San Jose, CA, 2023.

[19] Shen M., Xie J., Xu Y., Wang S., Cheng R., Fu W., Zhou Y., Tang H. X. (2024). Photonic
Link from Single-Flux-Quantum
Circuits to Room Temperature. Nat. Photonics.

[20] Bisang D., Horst Y., Thürig M., Menachery K., Koepfli S. M., Kohli M., De Leo E., Destraz M., Tedaldi V., Del Medico N., Hoessbacher C., Baeuerle B., Heni W., Leuthold J. (2024). Plasmonic
Modulators
in Cryogenic Environment Featuring Bandwidths in Excess of 100 GHz
and Reduced Plasmonic Losses. ACS Photonics.

[21] Van
Thiel T. C., Weaver M. J., Berto F., Duivestein P., Lemang M., Schuurman K. L., Žemlička M., Hijazi F., Bernasconi A. C., Ferrer C., Cataldo E., Lachman E., Field M., Mohan Y., De Vries F. K., Bultink C. C., Van Oven J. C., Mutus J. Y., Stockill R., Gröblacher S. (2025). Optical Readout of a Superconducting Qubit Using a
Piezo-Optomechanical Transducer. Nat. Phys..

[22] Wu H., Fu W., Feng M., Deppe D. (2021). 2.6 K VCSEL Data Link for Cryogenic
Computing. Appl. Phys. Lett..

[23] Imany P., Wang Z., DeCrescent R. A., Boutelle R. C., McDonald C. A., Autry T., Berweger S., Kabos P., Nam S. W., Mirin R. P., Silverman K. L. (2022). Quantum Phase Modulation with Acoustic
Cavities and Quantum Dots. Optica.

[24] Viheriälä, J. ; Uusitalo, T. ; Virtanen, H. ; Namvar, B. ; Rajala, P. ; Ranta, S. ; Hakkarainen, T. ; Tukiainen, A. ; Almuneau, G. ; Guina, M. Development of VCSELs for Cyogenic (4.2 K) Optical Interfaces. In 2023 IEEE Photonics Society Summer Topicals Meeting Series (SUM); IEEE: Sicily, Italy, 2023; pp 1–2..

[25] Lecocq F., Quinlan F., Cicak K., Aumentado J., Diddams S. A., Teufel J. D. (2021). Control and Readout of a Superconducting
Qubit Using a Photonic Link. Nature.

[26] Zhang Y. M., Borzenets V., Dubash N., Reynolds T., Wey Y. G., Bowers J. (1997). Cryogenic
Performance of a High-Speed GaInAs/InP p-i-n
Photodiode. J. Light. Technol..

[27] Julien-Neitzert D., Leung E. K., Islam N., Khorev S., Shekhar S., Chrostowski L., Young J. F., Salfi J. (2024). Cryogenic Optical-to-Microwave
Conversion Using Si Photonic Integrated Circuit Ge Photodiodes. APL Photonics.

[28] Nakamura T., Lee D., Horng J., Lecocq F., Teufel J., Quinlan F. (2025). Cryogenic
Photonic Link Using an Extended-InGaAs Photodiode and Short Pulse
Illumination toward High-Fidelity Drive of Superconducting Qubits. Optica Quantum.

[29] Mutum, S. ; Vliex, P. ; Bühler, J. ; Nielinger, D. ; Schlösser, M. ; Van Waasen, S. A Photonic Link at 4.7 K with > 1 GHz Bandwidth Towards an Optical Quantum Computing Interface. In 2025 IEEE/MTT-S International Microwave Symposium - IMS 2025; IEEE: San Francisco, CA, USA, 2025; pp 73–76..

[30] Gran, J. ; Malmbekk, H. ; Lind, K. Photodiodes as Current Source in High-Frequency Low Temperature Applications. In 29th Conference on Precision Electromagnetic Measurements (CPEM 2014); IEEE: Rio de Janeiro, Brazil, 2014; pp 266–267..

[31] Anferov A., Wan F., Harvey S. P., Simon J., Schuster D. I. (2025). Millimeter-Wave
Superconducting Qubit. PRX Quantum.

[32] Koepfli S. M., Baumann M., Koyaz Y., Gadola R., Güngör A., Keller K., Horst Y., Nashashibi S., Schwanninger R., Doderer M., Passerini E., Fedoryshyn Y., Leuthold J. (2023). Metamaterial Graphene Photodetector
with Bandwidth Exceeding 500 Gigahertz. Science.

[33] Landy N. I., Sajuyigbe S., Mock J. J., Smith D. R., Padilla W. J. (2008). Perfect
Metamaterial Absorber. Phys. Rev. Lett..

[34] Lochbaum A., Dorodnyy A., Koch U., Koepfli S. M., Volk S., Fedoryshyn Y., Wood V., Leuthold J. (2020). Compact Mid-Infrared
Gas Sensing Enabled by an All-Metamaterial Design. Nano Lett..

[35] Dorodnyy A., Koepfli S. M., Lochbaum A., Leuthold J. (2020). Design of
CMOS-Compatible
Metal–Insulator–Metal Metasurfaces via Extended Equivalent-Circuit
Analysis. Sci. Rep.

[36] Giovannetti G., Khomyakov P. A., Brocks G., Karpan V. M., van den
Brink J., Kelly P. J. (2008). Doping Graphene with Metal Contacts. Phys. Rev. Lett..

[37] Xia F., Mueller T., Golizadeh-Mojarad R., Freitag M., Lin Y., Tsang J., Perebeinos V., Avouris P. (2009). Photocurrent Imaging
and Efficient Photon Detection in a Graphene Transistor. Nano Lett..

[38] Mueller T., Xia F., Avouris P. (2010). Graphene Photodetectors for High-Speed Optical Communications. Nature Photon.

[39] Novoselov K. S., Geim A. K., Morozov S. V., Jiang D., Zhang Y., Dubonos S. V., Grigorieva I. V., Firsov A. A. (2004). Electric Field Effect
in Atomically Thin Carbon Films. Science.

[40] Mišeikis V., Marconi S., Giambra M. A., Montanaro A., Martini L., Fabbri F., Pezzini S., Piccinini G., Forti S., Terrés B., Goykhman I., Hamidouche L., Legagneux P., Sorianello V., Ferrari A. C., Koppens F. H. L., Romagnoli M., Coletti C. (2020). Ultrafast, Zero-Bias, Graphene Photodetectors
with Polymeric Gate Dielectric on Passive Photonic Waveguides. ACS Nano.

[41] Gungor A. C., Koepfli S. M., Baumann M., Ibili H., Smajic J., Leuthold J. (2022). Modeling Hydrodynamic Charge Transport in Graphene. Materials.

[42] Chen J.-H., Jang C., Xiao S., Ishigami M., Fuhrer M. S. (2008). Intrinsic
and Extrinsic Performance Limits of Graphene Devices on SiO2. Nat. Nanotechnol..

[43] Zhu W., Perebeinos V., Freitag M., Avouris P. (2009). Carrier Scattering,
Mobilities, and Electrostatic Potential in Monolayer, Bilayer, and
Trilayer Graphene. Phys. Rev. B:Condens. Matter
Mater. Phys..

[44] Srivastava P. K., Ghosh S. (2015). Defect Engineering
as a Versatile Route to Estimate Various Scattering
Mechanisms in Monolayer Graphene on Solid Substrates. Nanoscale.

[45] Urich A., Unterrainer K., Mueller T. (2011). Intrinsic Response Time of Graphene
Photodetectors. Nano Lett..

[46] Koepfli S. M., Baumann M., Gadola R., Nashashibi S., Koyaz Y., Rieben D., Güngör A. C., Doderer M., Keller K., Fedoryshyn Y., Leuthold J. (2024). Controlling Photothermoelectric Directional Photocurrents
in Graphene with over 400 GHz Bandwidth. Nat.
Commun..

[47] Graham M. W., Shi S.-F., Ralph D. C., Park J., McEuen P. L. (2013). Photocurrent
Measurements of Supercollision Cooling in Graphene. Nature Phys..

[48] Yoshioka K., Wakamura T., Hashisaka M., Watanabe K., Taniguchi T., Kumada N. (2022). Ultrafast Intrinsic Optical-to-Electrical Conversion
Dynamics in a Graphene Photodetector. Nat. Photonics.

[49] Ma Q., Gabor N. M., Andersen T. I., Nair N. L., Watanabe K., Taniguchi T., Jarillo-Herrero P. (2014). Competing Channels for Hot-Electron
Cooling in Graphene. Phys. Rev. Lett..

[50] Ma Q., Lui C. H., Song J. C. W., Lin Y., Kong J. F., Cao Y., Dinh T. H., Nair N. L., Fang W., Watanabe K., Taniguchi T., Xu S.-Y., Kong J., Palacios T., Gedik N., Gabor N. M., Jarillo-Herrero P. (2019). Giant Intrinsic
Photoresponse in Pristine Graphene. Nat. Nanotechnol..

[51] Dorgan V. E., Bae M.-H., Pop E. (2010). Mobility and
Saturation Velocity
in Graphene on SiO2. Appl. Phys. Lett..

[52] Song J. C. W., Levitov L. S. (2014). Shockley-Ramo Theorem and Long-Range Photocurrent Response
in Gapless Materials. Phys. Rev. B:Condens.
Matter Mater. Phys..

[53] Liu M., Pelton M., Guyot-Sionnest P. (2009). Reduced Damping
of Surface Plasmons
at Low Temperatures. Phys. Rev. B:Condens. Matter
Mater. Phys..

[54] Bouillard J.-S. G., Dickson W., O’Connor D. P., Wurtz G. A., Zayats A. V. (2012). Low-Temperature
Plasmonics of Metallic Nanostructures. Nano
Lett..

[55] Heni, W. ; Habegger, P. ; Leo, E. D. ; Destraz, M. ; Meier, N. ; Medico, N. D. ; Tedaldi, V. ; Funck, C. ; Langenbach, A. ; Duran, H. ; Guesken, N. A. ; Leuthold, J. ; Hoessbacher, C. ; Baeuerle, B. Plasmonic PICs Terabit Modulation on the Micrometer Scale. In European Conference on Optical Communication (ECOC) 2022; Optica Publishing Group, 2022..

[56] Lee J. E., Ahn G., Shim J., Lee Y. S., Ryu S. (2012). Optical Separation
of Mechanical Strain from Charge Doping in Graphene. Nat. Commun..

[57] Boscá A., Pedrós J., Martínez J., Palacios T., Calle F. (2016). Automatic
Graphene Transfer System for Improved Material Quality and Efficiency. Sci. Rep.

[58] Fischetti M. V., Neumayer D. A., Cartier E. A. (2001). Effective
Electron Mobility in Si
Inversion Layers in Metal–Oxide–Semiconductor Systems
with a High-κ Insulator: The Role of Remote Phonon Scattering. J. Appl. Phys..

[59] Muench J. E., Ruocco A., Giambra M. A., Miseikis V., Zhang D., Wang J., Watson H. F. Y., Park G. C., Akhavan S., Sorianello V., Midrio M., Tomadin A., Coletti C., Romagnoli M., Ferrari A. C., Goykhman I. (2019). Waveguide-Integrated,
Plasmonic Enhanced Graphene Photodetectors. Nano Lett..

[60] Ghahari F., Xie H.-Y., Taniguchi T., Watanabe K., Foster M. S., Kim P. (2016). Enhanced Thermoelectric Power in Graphene: Violation of the Mott
Relation by Inelastic Scattering. Phys. Rev.
Lett..

[61] Soundarapandian K. P., Montanaro A., Vangelidis I., Koepfli S. M., Orsini L., Ceccanti M., Kulmer L., Misal M., Reep T., Castilla S., Watanabe K., Taniguchi T., Tongay S. A., Thourhout D. V., Leuthold J., Tielrooij K.-J., Lidorikis E., Romagnoli M., Sorianello V., Koppens F. H. L. (2026). C-Band 160 Gbs-1
Zero-Bias Graphene Photodetectors:
Breaking the Responsivity-Bandwidth Trade-off by Heterostructure Engineering. arXiv.

